# The impact of aberrant lipid metabolism on the immune microenvironment of gastric cancer: a mini review

**DOI:** 10.3389/fimmu.2025.1639823

**Published:** 2025-08-18

**Authors:** Shuangyu Chen, Wenqian Chen, Tinghui Xu, Jiayang Li, Jianghao Yu, Yibo He, Shengliang Qiu

**Affiliations:** 1 The First Affiliated Hospital of Zhejiang Chinese Medical University (Zhejiang Provincial Hospital of Chinese Medicine), Hangzhou, Zhejiang, China; 2 School of Medical Technology and Information Engineering, Zhejiang Chinese Medical University, Hangzhou, Zhejiang, China

**Keywords:** gastric cancer, lipid metabolism, tumor immune microenvironment, CD8+ T cells, tumor-associated macrophages, immunotherapy resistance, fatty acid oxidation, immune checkpoint blockade

## Abstract

Gastric cancer (GC) remains one of the leading causes of cancer-related mortality worldwide, with limited responses to immune checkpoint blockade (ICB) therapies in most patients. Increasing evidence indicates that the tumor immune microenvironment (TIME) plays a crucial role in immunotherapy outcomes. Among various metabolic abnormalities in the TIME, dysregulated lipid metabolism has emerged as a critical determinant of immune cell fate, differentiation, and function. In this review, we comprehensively summarize the current understanding of the immune landscape in GC, focusing on how altered lipid metabolism reshapes immune cell populations—including tumor-associated macrophages (TAMs), dendritic cells (DCs), regulatory T cells (Tregs), myeloid-derived suppressor cells (MDSCs), and cytotoxic CD8^+^ T cells. We highlight key metabolic pathways such as fatty acid oxidation(FAO), cholesterol homeostasis, and lipid uptake that impact immune cell activity, contributing to immune evasion and therapeutic resistance. Importantly, we explore emerging therapeutic strategies targeting lipid metabolism, including inhibitors of cluster of differentiation 36 (CD36), fatty acid synthase (FASN), and sterol regulatory element-binding protein 1 (SREBP1) and discuss their synergistic potential when combined with ICB therapies. In conclusion, lipid metabolic reprogramming represents a promising yet underexplored axis in modulating antitumor immunity in GC. Integrating metabolic intervention with immunotherapy holds potential to overcome current treatment limitations and improve clinical outcomes. Future studies incorporating spatial omics and single-cell profiling will be essential to elucidate cell-type specific metabolic dependencies and foster translational breakthroughs.

## 1 Introduction

According to GLOBOCAN 2022 statistics, in 2022, more than 968,000 new cases of gastric cancer (GC) were added, with nearly 660,000 deaths, ranking fifth globally both in terms of incidence and mortality. The region with the highest incidence rate is East Asia, which imposes a significant burden on cancer (1). Consequently, an urgent exploration and development of new therapeutic approaches has become imperative.

The tumor microenvironment (TME) is a complex system that can inhibit immune responses while promoting tumor progression. The composition of the TME differs across different tumor types, but its defining features include immune cells, stromal cells, vasculature, and extracellular matrix (2, 3). The complexity and dynamic interactions within the TME contribute significantly to the aggressive nature of GC and the development of therapeutic resistance (4). Therefore, understanding the intricate characteristics of the TME, particularly metabolic reprogramming within this milieu, is of substantial clinical importance for developing effective treatments for GC patients.

Metabolic reprogramming is widely recognized as a hallmark of cancer, allowing tumor cells to sustain proliferation, evade immune surveillance, and survive under stressful conditions. Among various metabolic alterations, abnormal lipid metabolism has emerged as a pivotal player in cancer progression, influencing energy metabolism, membrane biosynthesis, and signaling pathways (5–7). Cancer cells undergo significant lipid metabolic reprogramming, including increased lipid uptake, enhanced fatty acid synthesis (FAS), and elevated fatty acid oxidation (FAO). These alterations not only provide essential metabolic substrates but also enable cancer cells to resist oxidative stress, promoting tumor survival and resistance to conventional therapies (8).

Key enzymes involved in lipid metabolism, such as fatty acid synthase (FASN), ATP citrate lyase (ACLY), and stearoyl-CoA desaturase (SCD), are upregulated in GC (9–11), indicating their potential as therapeutic targets. Aberrant lipid metabolic pathways influence the recruitment, differentiation, and function of key immune cell populations including tumor-associated macrophages (TAMs), regulatory T cells (Tregs),and myeloid-derived suppressor cells (MDSCs), dendritic cells (DCs), CD8+ T cells,contributing to an immunosuppressive microenvironment that facilitates tumor progression.

## 2 Lipid metabolic pathways and molecular mechanisms

A key energy-generating pathway in lipid metabolism is mitochondrial fatty acid β-oxidation, which is mediated by carnitine palmitoyl-transferase 1 (CPT1), especially the isoform CPT1a (12, 13). This enzyme facilitates the transport of long-chain fatty acids to the mitochondria for oxidative breakdown and ATP production, particularly under nutrient-deprived conditions (14). Simultaneously, cancer cells exploit exogenous lipid sources through dietary uptake, with cluster of differentiation 36 (CD36) functioning as a major fatty acid translocase (15). CD36 is frequently overexpressed in malignant cells, contributing to enhanced fatty acid uptake, intracellular lipid accumulation, and increased metabolic plasticity (16–18). This metabolic architecture is tightly regulated by oncogenic signaling cascades, especially the PI3K/Akt/mTOR axis. This axis activates sterol regulatory element-binding protein 1 (SREBP1), a master transcriptional regulator of lipid biosynthesis (19, 20). When SREBP1 is activated, the expression of key enzymes involved in fat production, such as FASN and acetyl CoA carboxylase (ACC), is enhanced. This can promote *de novo* fat generation and support the promotion of membrane biogenesis and proliferation (21, 22). The uptake of extracellular lipids via CD36 and the lipolysis-stimulated lipoprotein receptor (LSR) is often upregulated in tumors and is also responsive to PI3K/mTOR signaling, reinforcing the lipid supply for cancer progression (23–25). Enzymes like acyl-CoA synthetase long-chain family members (ACSLs) activate imported fatty acids and channel them into biosynthetic and storage pathways, while lipogenesis induced by SREBP1 inhibits ferroptosis and improves tumor cell survival (20, 26). Uptake of lipids by CD36 enhances metastatic potential and contributes to adaptation to the TME (27). Additionally, reorganization of lipid metabolism can alter antigen presentation and inhibit T-cell activation, leading to impairment of immune surveillance (28). Phospholipid remodeling represents another critical branch of lipid metabolism. This metabolic adaptation highlights the key function of lipid metabolism in coordinating cellular bioenergetics with tumor invasiveness and immune escape, laying the mechanistic foundation for its involvement in the formation of an immunosuppressive TME (29).

## 3 Overview of the immune microenvironment in gastric cancer

TME of GC is composed of various immune cell subsets and non-immune components, and is characterized by prominent immunosuppressive features. Single-cell analyses have revealed a highly heterogeneous pattern of immune cell infiltration within the TME of GC. Immunosuppressive components such as Tregs, MDSCs, and TAMs are widely distributed and are closely associated with ineffective antitumor immune responses (30–33). Tregs suppress CD8^+^ T cell activity and the antigen presentation process through multiple mechanisms, serving as key regulatory factors in the progression of GC (34, 35). MDSCs exacerbate the immunosuppressive state by secreting inhibitory factors and modulating macrophage polarization (36). Moreover, M2 polarization of TAMs in GC has been shown to be closely associated with immune evasion and poor prognosis (37–39). Another key mechanism underlying the immunosuppressive TME is the upregulation of immune checkpoints, such as PD-L1 and the CD39/CD73 axis, which inhibit T cell effector functions and promote tumor immune evasion (40, 41). Studies have indicated that the TME in GC patients often exhibits a “cold tumor” phenotype—characterized by low immune cell infiltration and weak immune activation—which not only predicts poor prognosis but also correlates with low responsiveness to immunotherapy (42, 43).

Immune infiltration patterns exhibit dynamic changes across different GC subtypes and treatment contexts. Neoadjuvant chemotherapy can significantly remodel the TME by enhancing CD8+T cell infiltration and reducing immunosuppressive cells, highlighting the plasticity of the immune landscape (44, 45). High-throughput analyses and multiplex immunofluorescence have revealed complex interactions among different immune cells within the TME, such as exosome-mediated communication between TAMs and cancer cells (46, 47).

Furthermore, the degree of immune cell infiltration is closely associated with clinical outcomes. For instance, high PD-L1 expression often coexists with an “immune-excluded” infiltration pattern, suggesting that patients may benefit from immune checkpoint inhibitor therapy (48, 49). Key molecular features of the TME significantly shape immune infiltration and immunotherapy responses in GC, highlighting new avenues for enhancing antitumor immunity (50–52). Among these features, spatial metabolic heterogeneity — particularly lipid gradients within the TME — has recently gained attention as a critical factor influencing immune cell behavior.

## 4 Interactions between aberrant lipid metabolism and immune cells

### 4.1 TAMs

TAMs, one of the most abundant immune cells in the GC immune microenvironment, exhibit significant metabolic plasticity. Under the stimulation of various cytokines, macrophages can be polarized into two phenotypes with different functions: M1 macrophages, which have pro-inflammatory and tumor-inhibiting effects; And M2 macrophages, which have anti-inflammatory and tumor-promoting effects Their functional state is closely linked to their lipid metabolic program. In gastric cancer, scavenger receptors such as CD36 mediate the endocytosis of fatty acids and cholesterol from the tumor microenvironment, leading to intracellular lipid accumulation and promoting the establishment of a highly immunosuppressive TME (53, 54). This process further activates the peroxisome proliferator-activated receptor γ (PPAR-γ) signaling pathway, upregulating FAO, promoting TAM towards a m2 polarized phenotype, and enhancing its oncogenic function (55, 56). Moreover, lipid uptake promotes enhanced FAO, providing a stable energy supply for M2-polarized TAMs and augmenting their secretion of immunosuppressive factors such as IL-10 and TGF-β (57–59). These alterations collectively contribute to the formation of a microenvironment that favors tumor survival and immune evasion (60, 61). Mechanistically, lipid uptake via CD36 facilitates intracellular fatty acid accumulation, which activates PPAR-γ signaling and upregulates key enzymes of FAO, such as CPT1A.

Further studies have revealed that the metabolic state of TAMs is a key determinant of their spatial distribution and functional heterogeneity. For example, lipid-rich TAMs are predominantly located in hypoxic regions, where they respond to tumor-derived factors such as IL-34 and signals associated with p53 inactivation, exhibiting enhanced immunosuppressive capabilities (62, 63). At the metabolic level, lipid metabolic reprogramming is closely regulated by the TRAF3/STAT6 pathway, which governs key transcriptional programs involved in the polarization process (64). Meanwhile, signaling molecules such as CD40 have been shown to promote the reprogramming of TAMs toward an antitumor phenotype by remodeling fatty acid and glutamine metabolism, highlighting the potential of metabolic interventions in reshaping TAM function (65). Overall, lipid uptake and metabolism determine the fate of TAMs, representing a critical regulatory axis within the GC immune microenvironment and a promising therapeutic target for future treatment strategies (66). These findings highlight the central role of TAM lipid metabolism in promoting immune evasion and progression of gastric cancer.

### 4.2 Dendritic cells

DCs within the GC immune microenvironment is often markedly suppressed by dysregulated lipid metabolism. In gastric cancer, this metabolic dysfunction contributes to impaired tumor antigen presentation and weakened immune surveillance.The lipid-rich tumor environment leads to lipid accumulation in DCs, particularly the formation of lipid droplets enriched with cholesterol and triglycerides, which significantly impairs their antigen-presenting capacity (67, 68). Lipid overload not only diminishes the expression of major histocompatibility complex (MHC) class I and II molecules but also suppresses the expression of costimulatory molecules such as CD80 and CD86, thereby limiting T cell activation (69, 70). Studies have shown that Epstein-Barr virus–associated GC exacerbates antigen presentation impairment by secreting exosomes that interfere with DC maturation (70). Moreover, tumor-induced lipid metabolic reprogramming can suppress mitochondrial function and glucose metabolism in DCs, driving them toward an immunotolerant phenotype (67, 68). A decline in cross-presentation capacity is another critical defect of lipid-laden DCs, particularly impairing their ability to elicit CD8^+^ T cell responses (71, 72). Some studies have reported that lipid accumulation hinders the ability of DCs to uptake and process extracellular antigens, thereby weakening their effectiveness in activating tumor-specific T cells (73, 74). Furthermore, Tregs form immunosuppressive complexes with DCs through a CXCR3-mediated chemotactic mechanism, further limiting the ability of DCs to activate CD8+ T cells (75). In recent years, engineered dendritic cell (DC) systems have been developed to bypass the metabolic impairments of natural DCs, offering new avenues for tumor vaccines and targeted immunotherapy (76, 77). Therefore, targeting lipid metabolic regulatory pathways is considered a potential strategy to restore DC immune function and enhance immune responses in gastric cancer (78, 79).

### 4.3 Tregs and MDSCs

Tregs are abundantly infiltrated in the GC immune microenvironment and rely on lipid metabolism to maintain their stability and immunosuppressive function. Studies have shown that within the tumor environment, Tregs gain an energetic advantage by enhancing FAO, which sustains their Foxp3 expression and suppressive capacity (80, 81). PD-1 deficiency disrupts the metabolic stability of Tregs, suggesting that their metabolic adaptability is a critical factor in the establishment of immune tolerance (80). Moreover, fatty acid-binding protein 5 (FABP5) and the SIRT1–CX3CL1 axis play important roles in regulating lipid metabolism in Tregs, influencing their distribution within the TME and their immunosuppressive capacity (82, 83). In lipid-rich microenvironments, Tregs exhibit enhanced stability and activity, representing one of the major obstacles to the efficacy of immune checkpoint inhibition therapy (84, 85).

Similar to Tregs, MDSCs exhibit potent immunosuppressive properties regulated by lipid metabolism. In high-lipid microenvironments, they sustain their survival through FAS and cholesterol metabolism, while secreting a range of immunosuppressive factors (29, 86). Ginger polysaccharide–induced lipid metabolic disruption can promote apoptosis of MDSCs, indicating that targeting lipid metabolism holds potential for enhancing immune responses (86). Within the GC TME, MDSCs cooperate with Tregs to establish a metabolically coupled immunosuppressive network (87, 88). Recent studies have shown that cancer-associated fibroblasts (CAFs) influence the metabolic activity of MDSCs through CD36 and the secretion of macrophage migration inhibitory factor (MIF), further exacerbating immune evasion (87). In summary, targeting lipid metabolism has emerged as a key strategy for modulating the functions of Tregs and MDSCs and overcoming immune tolerance (29, 85).

### 4.4 CD8^+^ T cells

CD8^+^ T cells are the central effector cells in antitumor immune responses, and their functional state is significantly influenced by dysregulated lipid metabolism within the TME. In the GC microenvironment, fatty acid uptake and cholesterol metabolism reshape the metabolic programming of CD8^+^ T cells, leading to metabolic imbalance, enhanced exhaustion phenotypes, and reduced cytotoxic function (89). Tumor cells secrete lipid metabolism–regulating factors such as SCD1 and FABP5, which elevate levels of free fatty acids and oxidized lipids in the TME. This induces the accumulation of reactive oxygen species (ROS) in CD8^+^ T cells, leading to lipid peroxidation and mitochondrial damage (90). This process is accompanied by the upregulation of inhibitory receptors such as PD-1 and TIGIT, ultimately leading to T cell exhaustion and the loss of sustained cytotoxic activity (91). Moreover, excess cholesterol can accumulate in the membranes of CD8^+^ T cells, disrupting immunological synapse formation and TCR signaling, thereby further suppressing their effector functions (92).

**Figure 1:**
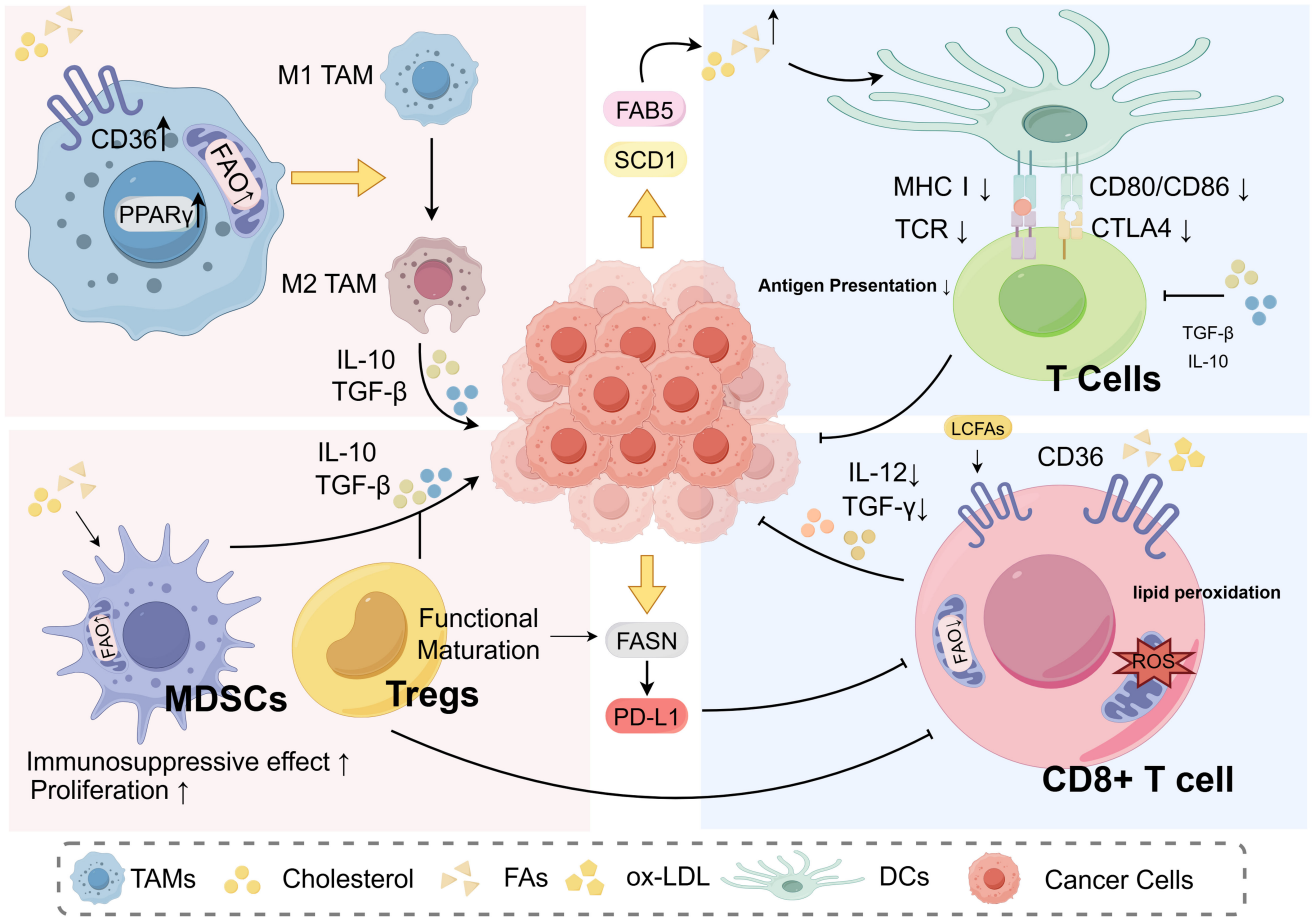
Interactions between aberrant lipid metabolism and immune cells.

Studies have also indicated that certain lipid metabolic pathways exert bidirectional regulatory effects on CD8^+^ T cells. Tissue-resident CD8^+^ T cells rely on FAO to sustain energy supply and long-term survival; however, in the nutrient-deprived and competitive TME, this metabolic dependency may actually constrain the sustained activation of their effector functions (89). Under high-lipid conditions, tumor cells compete with CD8^+^ T cells for nutritional substrates, leading to energy deprivation in CD8^+^ T cells. This results in a state of “functional starvation,” characterized by reduced expression of effector molecules such as Granzyme B and IFN-γ (27, 91). Therefore, targeting lipid metabolic pathways—such as CD36 inhibition, FAO blockade, or cholesterol metabolism modulation—is considered a promising strategy to restore CD8^+^ T cell function and enhance the efficacy of immunotherapy (90, 93) (**Figure 1**).

## 5 Clinical and therapeutic implications

Lipid metabolic reprogramming is not only a key mechanism in shaping the TME of GC, but also offers multidimensional therapeutic targets for clinical intervention. High expression of key lipid metabolic molecules such as CD36, FASN, and SREBP1 is closely associated with the infiltration of immunosuppressive cells and T-cell exhaustion, and is considered one of the major contributors to immunotherapy resistance (94–96). For instance, Li et al. found that lipid metabolic imbalance can promote symbiotic signaling pathways between CAFs and TAMs, which significantly impairs the efficacy of immune checkpoint inhibitors (ICIs) (97). Emerging lipid-targeted strategies—such as FASN inhibitors, FAO pathway blockers, and cholesterol metabolism modulators—are being actively explored to enhance CD8^+^T cell function, inhibit TAM polarization, and reduce Treg-mediated immunosuppression (94, 98, 99). Moreover, lipid metabolism–related genes have also been identified as potential predictive biomarkers of immune response. Genes such as RGS2, APOD, and MTTP have demonstrated promising prognostic and therapeutic response prediction value in multiple studies (94, 96, 98).

Combination therapy strategies are emerging as a key approach to overcoming the bottlenecks of immunotherapy in GC Several clinical trials—such as ATTRACTION-2, ATTRACTION-4, KEYNOTE-859, KEYNOTE-061 and CheckMate-649—have validated the efficacy of combining ICIs with chemotherapy (100–104). Combination strategies involving CD36 antagonists or cholesterol synthase inhibitors have significantly enhanced antitumor immune responses in preclinical models (94). Meanwhile, lipid metabolism–based immune subtyping approaches are increasingly being employed to guide the selection of GC patients for immunotherapy (101, 105). In summary, the role of lipid metabolism in precision immunotherapy for GC is becoming increasingly prominent. Existing clinical trials combining immune checkpoint inhibitors with chemotherapy have demonstrated heterogeneous outcomes, which may partially reflect underlying metabolic states of the tumor immune microenvironment (106–110). Aberrant expression of lipid metabolism–related molecules such as FASN, CD36, and SREBP1 has been associated with immune cell exhaustion, Treg enrichment, and impaired dendritic cell function, suggesting their potential value as both therapeutic targets and predictive biomarkers (111–114). Integrating lipidomic analysis into future clinical trial designs may enhance stratification strategies and optimize combination regimens to overcome resistance (**Table 1**).

**Table 1:** Clinical trials of immunotherapy-based combination strategies in gastric cancer.

Trial	Phase	Drugs	Actual enrollment	Study period	Reference	Lipid metabolism/immune remodeling findings
NCT02872116 (CHECKMATE-649)	III	Nivolumab + Ipilimumab or Nivolumab in Combination With Oxaliplatin + Fluoropyrimidine vs Oxaliplatin + Fluoropyrimidine	2031	May 27, 2020- May 31, 2024	(100)	↑ CD8^+^ T cells, ↓ PD-L1 immune evasion; lipid modifications regulate PD-L1.
NCT02746796 (ATTRCTION-04)	II/III	SOX/Capecitabine + Oxaliplatin with vs without Nivolumab	724	March 7, 2017 – May 10, 2018	(101)	↑ CD8^+^ T cells; enhanced tumor microenvironment immune activation.
NCT03675737 (KEYNOTE-859)	III	Pembrolizumab+ Chemotherapy vs Placebo + Chemotherapy	1579	November 8, 2018 – September 28, 2024	(102)	↑ PD-L1 expression, immune activation linked to lipid gene co-signatures.
NCT03878472	II	Camrelizumab + Apatinib + S-1 ± Oxaliplatin	25	April 1, 2019 – May 31, 2024	(105)	↑ CD8^+^ T cells, ↓ PD-L1 immune evasion.
NCT04082364 (MAHOGANY)	II/III	Combination Margetuximab, Retifanlimab, Tebotelimab, and Chemotherapy	81	September 30, 2019 - December 2023	(106)	↑ T-cell activation via PD-1 and LAG-3 blockade; HER2–PD-L1 immune crosstalk implicated.
NCT03335540 (ADVISE)	I	Nivolumab + Ipilimumab vs Nivolumab	20	May 7, 2018 – August 25, 2021	(107)	↑ Immune markers in low/intermediate PD-L1 tumors; ↑ T-cell and macrophage activation.
NCT03662659 (RELATIVITY-060)	II	Relatlimab + Nivolumab + XELOX/FOLFOX/SOX vs. Nivolumab + XELOX/FOLFOX/SOX	274	October 16, 2018 – January 16, 2024	(108)	↑ T-cell activation via PD-1 and LAG-3 blockade
NCT04908566	II	PD-1 inhibitor + mFOLFIRINOX vs. mFOLFIRINOX	30	August 2023 – May 2025	(109)	↑ CD8^+^ T and NK cells, ↓ macrophages and FOXP3^+^ Tregs; dynamic immune remodeling predicts response
NCT04997837	III	Chemotherapy + PD-1 inhibitor + Radiotherapy VS Chemotherapy	433	July 21, 2021 – July 21, 2027	(110)	Radiation-induced PD-L1 upregulation
NCT03615326 (KEYNOTE-811)	III	Pembrolizumab/Trastuzumab/Chemotherapy vs Trastuzumab/Chemotherapy	698	October 5, 2018 - March 20, 2024	(111)	↑ T-cell activation; HER2–PD-L1 crosstalk enhances immune response with pembrolizumab.
NCT02589496	II	Pembrolizumab	45	March 26, 2016 – December 2021	(113)	↑ Immune activation; metabolism pathways and epigenetic features linked to tumor microenvironment score (TMEscore) predicting ICB response.
NCT04182724 (KEYNOTE-061)	II	PD-1 inhibitor + albumin-bound paclitaxel + apatinib	43	July 11, 2019 – October 13, 2022	(114)	↑ PD-L1 expression; VEGFR inhibition and immune activation via PD-1 blockade.
NCT02267343 (ATTRACTION-2)	III	Nivolumab vs Placebo	493	October 2014 – January 2021	(115)	↑ PD-L1–dependent immune response; lipid metabolism not reported.
NCT05008783	III	Cadonilimab + Oxaliplatin + Capecitabine (XELOX) vs. Placebo + Oxaliplatin + Capecitabine (XELOX)	610	September 17, 2021 – October 18, 2025	(116)	↑ PD-L1 expression; enhanced immune activation via dual PD-1/CTLA-4 blockade.

↑, upregulated; ↓, downregulated.

## 6 Research gaps and future perspectives

Although the role of lipid metabolism in regulating the immune microenvironment of GC has been progressively elucidated, many gaps remain in understanding its mechanistic network. Current research primarily focuses on classical lipid metabolism regulators such as CD36 and FASN, while the roles of non-coding RNAs and RNA modifications (e.g. m^6^A) in the cross-regulation of lipid metabolism remain largely underexplored (115–117). Moreover, how lipid metabolism specifically affects different immune cell subsets—such as tissue-resident memory T cells (Trm) and progenitor-exhausted T cells (Tpex)—remains insufficiently investigated at the single-cell resolution level (118, 119). Most current mechanistic studies are based on *in vitro* cell experiments and traditional animal models, with a lack of application of emerging technologies—such as spatial transcriptomics, spatial metabolomics, and single-cell lipidomics—for constructing a “functional lipid map” within the immune microenvironment (120, 121).

In future research, a primary focus should be the expanded systematic screening of lipid metabolism regulators, including transporters, enzymes, and intermediate metabolites, to evaluate their immunological effects (122, 123). Secondly, integrating clinical cohorts to perform lipid metabolic phenotyping and establishing a biomarker system capable of predicting immunotherapy response and resistance risk will be critical for advancing personalized treatment (124–126). Moreover, constructing *in vitro* microenvironment models—such as organoid–immune cell co-culture systems—or developing novel drug delivery platforms targeting lipid metabolism will help bridge the gap between basic research and clinical application in metabolic immune regulation (127). Building on this foundation, conducting multicenter prospective clinical studies to evaluate the efficacy and safety of lipid metabolism–targeted interventions combined with immunotherapy will be a key pathway toward the clinical translation of metabolism-based immunotherapies (128, 129).

## 7 Conclusion

Lipid metabolism plays a central regulatory role in the TME of GC. Lipid competition between tumor cells and immune cells not only reshapes energy metabolism patterns but also alters immune cell functional states, inducing immunosuppressive phenotypes such as M2 polarization of TAMs, impaired antigen presentation by DCs, enhanced Treg functionality, and exhaustion of CD8^+^ T cells (30, 32, 34). Lipid metabolic reprogramming mechanisms—including CD36-mediated lipid uptake, enhanced FAO, and cholesterol accumulation—have been shown to play critical roles in GC progression and immune evasion by regulating immune checkpoint expression, immune cell metabolic adaptation, and the secretion of immunosuppressive factors (40, 48, 101). Targeting lipid metabolic pathways—such as FASN, CPT1A, CD36, or cholesterol metabolism—can enhance immunotherapeutic responses and alleviate the immunosuppressive nature of the TME, demonstrating promising translational potential (123). However, the cell-specific functions of lipid metabolism across different immune cell subsets, its spatial heterogeneity, and the interplay between metabolic and epigenetic regulation axes remain to be further investigated (119, 130, 131). Future research should integrate emerging technologies such as spatial transcriptomics, single-cell lipidomics, and multi-omics analyses, while establishing clinical cohorts to explore predictive biomarkers and novel strategies for metabolism-targeted therapies (127, 132).

## References

[1] Bray F Laversanne M Sung H Ferlay J Siegel RL Soerjomataram I . Global cancer statistics 2022: GLOBOCAN estimates of incidence and mortality worldwide for 36 cancers in 185 countries. CA: Cancer J Clin. (2024) 74:229–63. doi: 10.3322/caac.21834, PMID: 38572751

[2] Anderson NM Simon MC . The tumor microenvironment. Curr biol: CB. (2020) 30:R921–r5. doi: 10.1016/j.cub.2020.06.081, PMID: 32810447 PMC8194051

[3] Yasuda T Wang YA . Gastric cancer immunosuppressive microenvironment heterogeneity: implications for therapy development. Trends cancer. (2024) 10:627–42. doi: 10.1016/j.trecan.2024.03.008, PMID: 38600020 PMC11292672

[4] Liu Y Li C Lu Y Liu C Yang W . Tumor microenvironment-mediated immune tolerance in development and treatment of gastric cancer. Front Immunol. (2022) 13:1016817. doi: 10.3389/fimmu.2022.1016817, PMID: 36341377 PMC9630479

[5] Snaebjornsson MT Janaki-Raman S Schulze A . Greasing the wheels of the cancer machine: the role of lipid metabolism in cancer. Cell Metab. (2020) 31:62–76. doi: 10.1016/j.cmet.2019.11.010, PMID: 31813823

[6] Broadfield LA Pane AA Talebi A Swinnen JV Fendt SM . Lipid metabolism in cancer: New perspectives and emerging mechanisms. Dev Cell. (2021) 56:1363–93. doi: 10.1016/j.devcel.2021.04.013, PMID: 33945792

[7] Jin HR Wang J Wang ZJ Xi MJ Xia BH Deng K . Lipid metabolic reprogramming in tumor microenvironment: from mechanisms to therapeutics. J Hematol Oncol. (2023) 16:103. doi: 10.1186/s13045-023-01498-2, PMID: 37700339 PMC10498649

[8] Bacci M Lorito N Smiriglia A Morandi A . Fat and furious: lipid metabolism in antitumoral therapy response and resistance. Trends cancer. (2021) 7:198–213. doi: 10.1016/j.trecan.2020.10.004, PMID: 33281098

[9] El-Rifai W Frierson HF Jr. Moskaluk CA Harper JC Petroni GR Bissonette EA . Genetic differences between adenocarcinomas arising in Barrett’s esophagus and gastric mucosa. Gastroenterology. (2001) 121:592–8. doi: 10.1053/gast.2001.27215, PMID: 11522743

[10] Ezzeddini R Taghikhani M Somi MH Samadi N Rasaee MJ . Clinical importance of FASN in relation to HIF-1α and SREBP-1c in gastric adenocarcinoma. Life Sci. (2019) 224:169–76. doi: 10.1016/j.lfs.2019.03.056, PMID: 30914315

[11] Gao Y Li J Xi H Cui J Zhang K Zhang J . Stearoyl-CoA-desaturase-1 regulates gastric cancer stem-like properties and promotes tumour metastasis via Hippo/YAP pathway. Br J cancer. (2020) 122:1837–47. doi: 10.1038/s41416-020-0827-5, PMID: 32350414 PMC7283337

[12] Wang J Xiang H Lu Y Wu T Ji G . The role and therapeutic implication of CPTs in fatty acid oxidation and cancers progression. Am J Cancer Res. (2021) 11:2477–94., PMID: 34249411 PMC8263643

[13] Wang C Zhang C Li X Shen J Xu Y Shi H . CPT1A-mediated succinylation of S100A10 increases human gastric cancer invasion. J Cell Mol Med. (2019) 23:293–305. doi: 10.1111/jcmm.13920, PMID: 30394687 PMC6307794

[14] Terry AR Hay N . Emerging targets in lipid metabolism for cancer therapy. Trends Pharmacol Sci. (2024) 45:537–51. doi: 10.1016/j.tips.2024.04.007, PMID: 38762377 PMC11162322

[15] Glatz JFC Heather LC Luiken J . CD36 as a gatekeeper of myocardial lipid metabolism and therapeutic target for metabolic disease. Physiol Rev. (2024) 104:727–64. doi: 10.1152/physrev.00011.2023, PMID: 37882731

[16] Guerrero-Rodríguez SL Mata-Cruz C Pérez-Tapia SM Velasco-Velázquez MA . Role of CD36 in cancer progression, stemness, and targeting. Front Cell Dev Biol. (2022) 10:1079076. doi: 10.3389/fcell.2022.1079076, PMID: 36568966 PMC9772993

[17] Terry AR Nogueira V Rho H Ramakrishnan G Li J Kang S . CD36 maintains lipid homeostasis via selective uptake of monounsaturated fatty acids during matrix detachment and tumor progression. Cell Metab. (2023) 35:2060–76.e9. doi: 10.1016/j.cmet.2023.09.012, PMID: 37852255 PMC11748917

[18] Pascual G Avgustinova A Mejetta S Martín M Castellanos A Attolini CS . Targeting metastasis-initiating cells through the fatty acid receptor CD36. Nature. (2017) 541:41–5. doi: 10.1038/nature20791, PMID: 27974793

[19] Yi J Zhu J Wu J Thompson CB Jiang X . Oncogenic activation of PI3K-AKT-mTOR signaling suppresses ferroptosis via SREBP-mediated lipogenesis. Proc Natl Acad Sci United States America. (2020) 117:31189–97. doi: 10.1073/pnas.2017152117, PMID: 33229547 PMC7733797

[20] Chen M Li H Zheng S Shen J Chen Y Li Y . Nobiletin targets SREBP1/ACLY to induce autophagy-dependent cell death of gastric cancer cells through PI3K/Akt/mTOR signaling pathway. Phytomed: Int J phytother phytopharmacol. (2024) 128:155360. doi: 10.1016/j.phymed.2024.155360, PMID: 38547624

[21] Chen J Ye M Gu D Yu P Xu L Xue B . FTO-induced APOE promotes the Malignant progression of pancreatic neuroendocrine neoplasms through FASN-mediated lipid metabolism. Int J Biol Sci. (2025) 21:1478–96. doi: 10.7150/ijbs.103428, PMID: 39990672 PMC11844274

[22] Zhou X Li Y Yang C Chen D Wang T Liu T . Cordycepin reprogramming lipid metabolism to block metastasis and EMT via ERO1A/mTOR/SREBP1 axis in cholangiocarcinoma. Life Sci. (2023) 327:121698. doi: 10.1016/j.lfs.2023.121698, PMID: 37080351

[23] Kawabata K Takahashi T Tanaka K Kurokawa Y Yamamoto K Saito T . Lipolysis-stimulated lipoprotein receptor promote lipid uptake and fatty acid oxidation in gastric cancer. Gastric Cancer. (2024) 27:1258–72. doi: 10.1007/s10120-024-01552-z, PMID: 39294388

[24] Yang H Zhao H Ren Z Yi X Zhang Q Yang Z . Overexpression CPT1A reduces lipid accumulation via PPARα/CD36 axis to suppress the cell proliferation in ccRCC. Acta Biochim Biophys Sinica. (2022) 54:220–31. doi: 10.3724/abbs.2021023, PMID: 35130611 PMC9909300

[25] Zhao S Cheng L Shi Y Li J Yun Q Yang H . MIEF2 reprograms lipid metabolism to drive progression of ovarian cancer through ROS/AKT/mTOR signaling pathway. Cell Death disease. (2021) 12:18. doi: 10.1038/s41419-020-03336-6, PMID: 33414447 PMC7791105

[26] Zheng YN Lou SY Lu J Zheng FL Tang YM Zhang EJ . Selective PI3Kδ inhibitor TYM-3–98 suppresses AKT/mTOR/SREBP1-mediated lipogenesis and promotes ferroptosis in KRAS-mutant colorectal cancer. Cell Death disease. (2024) 15:474. doi: 10.1038/s41419-024-06848-7, PMID: 38956060 PMC11220027

[27] Tang Y Chen Z Zuo Q Kang Y . Regulation of CD8+ T cells by lipid metabolism in cancer progression. Cell Mol Immunol. (2024) 21:1215–30. doi: 10.1038/s41423-024-01224-z, PMID: 39402302 PMC11527989

[28] Wang Y Guo Z Isah AD Chen S Ren Y Cai H . Lipid metabolism and tumor immunotherapy. Front Cell Dev Biol. (2023) 11:1187989. doi: 10.3389/fcell.2023.1187989, PMID: 37261073 PMC10228657

[29] Zhang H Li S Wang D Liu S Xiao T Gu W . Metabolic reprogramming and immune evasion: the interplay in the tumor microenvironment. biomark Res. (2024) 12:96. doi: 10.1186/s40364-024-00646-1, PMID: 39227970 PMC11373140

[30] Chen J Liu K Luo Y Kang M Wang J Chen G . Single-cell profiling of tumor immune microenvironment reveals immune irresponsiveness in gastric signet-ring cell carcinoma. Gastroenterology. (2023) 165:88–103. doi: 10.1053/j.gastro.2023.03.008, PMID: 36921674

[31] Kang B Camps J Fan B Jiang H Ibrahim MM Hu X . Parallel single-cell and bulk transcriptome analyses reveal key features of the gastric tumor microenvironment. Genome Biol. (2022) 23:265. doi: 10.1186/s13059-022-02828-2, PMID: 36550535 PMC9773611

[32] Li Y Hu X Lin R Zhou G Zhao L Zhao D . Single-cell landscape reveals active cell subtypes and their interaction in the tumor microenvironment of gastric cancer. Theranostics. (2022) 12:3818–33. doi: 10.7150/thno.71833, PMID: 35664061 PMC9131288

[33] Zhou X Yang J Lu Y Ma Y Meng Y Li Q . Relationships of tumor differentiation and immune infiltration in gastric cancers revealed by single-cell RNA-seq analyses. Cell Mol Life sciences: CMLS. (2023) 80:57. doi: 10.1007/s00018-023-04702-1, PMID: 36729271 PMC9894979

[34] Negura I Pavel-Tanasa M Danciu M . Regulatory T cells in gastric cancer: Key controllers from pathogenesis to therapy. Cancer Treat Rev. (2023) 120:102629. doi: 10.1016/j.ctrv.2023.102629, PMID: 37769435

[35] Su J Mao X Wang L Chen Z Wang W Zhao C . Lactate/GPR81 recruits regulatory T cells by modulating CX3CL1 to promote immune resistance in a highly glycolytic gastric cancer. Oncoimmunology. (2024) 13:2320951. doi: 10.1080/2162402X.2024.2320951, PMID: 38419759 PMC10900271

[36] Tsutsumi C Ohuchida K Katayama N Yamada Y Nakamura S Okuda S . Tumor-infiltrating monocytic myeloid-derived suppressor cells contribute to the development of an immunosuppressive tumor microenvironment in gastric cancer. Gastric Cancer. (2024) 27:248–62. doi: 10.1007/s10120-023-01456-4, PMID: 38217732

[37] Zhang C Wei S Dai S Li X Wang H Zhang H . The NR_109/FUBP1/c-Myc axis regulates TAM polarization and remodels the tumor microenvironment to promote cancer development. J immunother Cancer. (2023) 11. doi: 10.1136/jitc-2022-006230, PMID: 37217247 PMC10230994

[38] Li Y Jiang L Chen Y Li Y Yuan J Lu J . Specific lineage transition of tumor-associated macrophages elicits immune evasion of ascitic tumor cells in gastric cancer with peritoneal metastasis. Gastric Cancer. (2024) 27:519–38. doi: 10.1007/s10120-024-01486-6, PMID: 38460015 PMC11016508

[39] He Y Hong Q Chen S Zhou J Qiu S . Reprogramming tumor-associated macrophages in gastric cancer: a pathway to enhanced immunotherapy. Front Immunol. (2025) 16:1558091. doi: 10.3389/fimmu.2025.1558091, PMID: 40098971 PMC11911521

[40] Miao Z Li J Wang Y Shi M Gu X Zhang X . Hsa_circ_0136666 stimulates gastric cancer progression and tumor immune escape by regulating the miR-375/PRKDC Axis and PD-L1 phosphorylation. Mol cancer. (2023) 22:205. doi: 10.1186/s12943-023-01883-y, PMID: 38093288 PMC10718020

[41] Huang T Ren X Tang X Wang Y Ji R Guo Q . Current perspectives and trends of CD39-CD73-eAdo/A2aR research in tumor microenvironment: a bibliometric analysis. Front Immunol. (2024) 15:1427380. doi: 10.3389/fimmu.2024.1427380, PMID: 39188712 PMC11345151

[42] Khan M Lin J Wang B Chen C Huang Z Tian Y . A novel necroptosis-related gene index for predicting prognosis and a cold tumor immune microenvironment in stomach adenocarcinoma. Front Immunol. (2022) 13:968165. doi: 10.3389/fimmu.2022.968165, PMID: 36389725 PMC9646549

[43] Lv K Sun M Fang H Wang J Lin C Liu H . Targeting myeloid checkpoint Siglec-10 reactivates antitumor immunity and improves anti-programmed cell death 1 efficacy in gastric cancer. J immunother Cancer. (2023) 11. doi: 10.1136/jitc-2023-007669, PMID: 37935567 PMC10649907

[44] Xing X Shi J Jia Y Dou Y Li Z Dong B . Effect of neoadjuvant chemotherapy on the immune microenvironment in gastric cancer as determined by multiplex immunofluorescence and T cell receptor repertoire analysis. J immunother Cancer. (2022) 10. doi: 10.1136/jitc-2021-003984, PMID: 35361730 PMC8971786

[45] Huan X Zou K Zhang P Ding H Luo C Xiang C . Neoadjuvant chemotherapy is linked to an amended anti-tumorigenic microenvironment in gastric cancer. Int immunopharmacol. (2024) 127:111352. doi: 10.1016/j.intimp.2023.111352, PMID: 38091833

[46] Qiu Y Lu G Li N Hu Y Tan H Jiang C . Exosome-mediated communication between gastric cancer cells and macrophages: implications for tumor microenvironment. Front Immunol. (2024) 15:1327281. doi: 10.3389/fimmu.2024.1327281, PMID: 38455041 PMC10917936

[47] Groen-van Schooten TS Franco Fernandez R van Grieken NCT Bos EN Seidel J Saris J . Mapping the complexity and diversity of tertiary lymphoid structures in primary and peritoneal metastatic gastric cancer. J immunother Cancer. (2024) 12. doi: 10.1136/jitc-2024-009243, PMID: 38955417 PMC11218001

[48] Zhao Q Yu H Shi M Wang X Fan Z Wang Z . Tumor microenvironment characteristics of lipid metabolism reprogramming related to ferroptosis and EndMT influencing prognosis in gastric cancer. Int immunopharmacol. (2024) 137:112433. doi: 10.1016/j.intimp.2024.112433, PMID: 38870879

[49] Zhong M Yu Z Wu Q Lu B Sun P Zhang X . PCDHGA10 as a potential prognostic biomarker and correlated with immune infiltration in gastric cancer. Front Immunol. (2024) 15:1500478. doi: 10.3389/fimmu.2024.1500478, PMID: 39687617 PMC11647002

[50] Lin Y Jing X Chen Z Pan X Xu D Yu X . Histone deacetylase-mediated tumor microenvironment characteristics and synergistic immunotherapy in gastric cancer. Theranostics. (2023) 13:4574–600. doi: 10.7150/thno.86928, PMID: 37649598 PMC10465215

[51] Bos J Groen-van Schooten TS Brugman CP Jamaludin FS van Laarhoven HWM Derks S . The tumor immune composition of mismatch repair deficient and Epstein-Barr virus-positive gastric cancer: A systematic review. Cancer Treat Rev. (2024) 127:102737. doi: 10.1016/j.ctrv.2024.102737, PMID: 38669788

[52] Li X Wu N Wang C Pei B Ma X Xie J . NALCN expression is down-regulated and associated with immune infiltration in gastric cancer. Front Immunol. (2025) 16:1512107. doi: 10.3389/fimmu.2025.1512107, PMID: 40013144 PMC11860897

[53] Li C Zhang L Qiu Z Deng W Wang W . Key molecules of fatty acid metabolism in gastric cancer. Biomolecules. (2022) 12:706. doi: 10.3390/biom12050706, PMID: 35625633 PMC9138239

[54] Aoki T Kinoshita J Munesue S Hamabe-Horiike T Yamaguchi T Nakamura Y . Hypoxia-induced CD36 expression in gastric cancer cells promotes peritoneal metastasis via fatty acid uptake. Ann Surg Oncol. (2023) 30:3125–36. doi: 10.1245/s10434-022-12465-5, PMID: 36042102 PMC10085939

[55] Liu S Zhang H Li Y Zhang Y Bian Y Zeng Y . S100A4 enhances protumor macrophage polarization by control of PPAR-γ-dependent induction of fatty acid oxidation. J immunother Cancer. (2021) 9. doi: 10.1136/jitc-2021-002548, PMID: 34145030 PMC8215236

[56] Zeng W Li F Jin S Ho PC Liu PS Xie X . Functional polarization of tumor-associated macrophages dictated by metabolic reprogramming. J Exp Clin Cancer research: CR. (2023) 42:245. doi: 10.1186/s13046-023-02832-9, PMID: 37740232 PMC10517486

[57] Gao J Liang Y Wang L . Shaping polarization of tumor-associated macrophages in cancer immunotherapy. Front Immunol. (2022) 13:888713. doi: 10.3389/fimmu.2022.888713, PMID: 35844605 PMC9280632

[58] Chen D Zhang X Li Z Zhu B . Metabolic regulatory crosstalk between tumor microenvironment and tumor-associated macrophages. Theranostics. (2021) 11:1016–30. doi: 10.7150/thno.51777, PMID: 33391518 PMC7738889

[59] Pan Y Yu Y Wang X Zhang T . Tumor-associated macrophages in tumor immunity. Front Immunol. (2020) 11:583084. doi: 10.3389/fimmu.2020.583084, PMID: 33365025 PMC7751482

[60] Li C Xu X Wei S Jiang P Xue L Wang J . Tumor-associated macrophages: potential therapeutic strategies and future prospects in cancer. J immunother Cancer. (2021) 9. doi: 10.1136/jitc-2020-001341, PMID: 33504575 PMC8728363

[61] Chu X Tian Y Lv C . Decoding the spatiotemporal heterogeneity of tumor-associated macrophages. Mol cancer. (2024) 23:150. doi: 10.1186/s12943-024-02064-1, PMID: 39068459 PMC11282869

[62] Nian Z Dou Y Shen Y Liu J Du X Jiang Y . Interleukin-34-orchestrated tumor-associated macrophage reprogramming is required for tumor immune escape driven by p53 inactivation. Immunity. (2024) 57:2344–61.e7. doi: 10.1016/j.immuni.2024.08.015, PMID: 39321806

[63] Liang Y Bu Q You W Zhang R Xu Z Gan X . Single-cell analysis reveals hypoxia-induced immunosuppressive microenvironment in intrahepatic cholangiocarcinoma. Biochim Biophys Acta Mol basis disease. (2024) 1870:167276. doi: 10.1016/j.bbadis.2024.167276, PMID: 38844114

[64] Shi JH Liu LN Song DD Liu WW Ling C Wu FX . TRAF3/STAT6 axis regulates macrophage polarization and tumor progression. Cell Death differentiation. (2023) 30:2005–16. doi: 10.1038/s41418-023-01194-1, PMID: 37474750 PMC10406838

[65] Liu PS Chen YT Li X Hsueh PC Tzeng SF Chen H . CD40 signal rewires fatty acid and glutamine metabolism for stimulating macrophage anti-tumorigenic functions. Nat Immunol. (2023) 24:452–62. doi: 10.1038/s41590-023-01430-3, PMID: 36823405 PMC9977680

[66] Xiang X Wang J Lu D Xu X . Targeting tumor-associated macrophages to synergize tumor immunotherapy. Signal transduction targeted Ther. (2021) 6:75. doi: 10.1038/s41392-021-00484-9, PMID: 33619259 PMC7900181

[67] Sun Z Zhang L Liu L . Reprogramming the lipid metabolism of dendritic cells in tumor immunomodulation and immunotherapy. Biomed pharmacother = Biomed pharmacotherapie. (2023) 167:115574. doi: 10.1016/j.biopha.2023.115574, PMID: 37757492

[68] Peng X He Y Huang J Tao Y Liu S . Metabolism of dendritic cells in tumor microenvironment: for immunotherapy. Front Immunol. (2021) 12:613492. doi: 10.3389/fimmu.2021.613492, PMID: 33732237 PMC7959811

[69] Chen J Duan Y Che J Zhu J . Dysfunction of dendritic cells in tumor microenvironment and immunotherapy. Cancer Commun (London England). (2024) 44:1047–70. doi: 10.1002/cac2.12596, PMID: 39051512 PMC11492303

[70] Hinata M Kunita A Abe H Morishita Y Sakuma K Yamashita H . Exosomes of epstein-barr virus-associated gastric carcinoma suppress dendritic cell maturation. Microorganisms. (2020) 8:1776. doi: 10.3390/microorganisms8111776, PMID: 33198173 PMC7697542

[71] MacNabb BW Tumuluru S Chen X Godfrey J Kasal DN Yu J . Dendritic cells can prime anti-tumor CD8(+) T cell responses through major histocompatibility complex cross-dressing. Immunity. (2022) 55:982–97.e8. doi: 10.1016/j.immuni.2022.04.016, PMID: 35617964 PMC9883788

[72] Murphy TL Murphy KM . Dendritic cells in cancer immunology. Cell Mol Immunol. (2022) 19:3–13. doi: 10.1038/s41423-021-00741-5, PMID: 34480145 PMC8752832

[73] Hilligan KL Ronchese F . Antigen presentation by dendritic cells and their instruction of CD4+ T helper cell responses. Cell Mol Immunol. (2020) 17:587–99. doi: 10.1038/s41423-020-0465-0, PMID: 32433540 PMC7264306

[74] Chudnovskiy A Castro TBR Nakandakari-Higa S Cui A Lin CH Sade-Feldman M . Proximity-dependent labeling identifies dendritic cells that drive the tumor-specific CD4(+) T cell response. Sci Immunol. (2024) 9:eadq8843. doi: 10.1126/sciimmunol.adq8843, PMID: 39365874 PMC12419230

[75] Moreno Ayala MA Campbell TF Zhang C Dahan N Bockman A Prakash V . CXCR3 expression in regulatory T cells drives interactions with type I dendritic cells in tumors to restrict CD8(+) T cell antitumor immunity. Immunity. (2023) 56:1613–30.e5. doi: 10.1016/j.immuni.2023.06.003, PMID: 37392735 PMC10752240

[76] Mateus D Sebastião AI Frasco MF Carrascal MA Falcão A Gomes CM . Artificial dendritic cells: A new era of promising antitumor immunotherapy. Small (Weinheim an der Bergstrasse Germany). (2023) 19:e2303940. doi: 10.1002/smll.202303940, PMID: 37469192

[77] Zheng J Wang M Pang L Wang S Kong Y Zhu X . Identification of a novel DEC-205 binding peptide to develop dendritic cell-targeting nanovaccine for cancer immunotherapy. J Controlled release. (2024) 373:568–82. doi: 10.1016/j.jconrel.2024.07.056, PMID: 39067792

[78] Marciscano AE Anandasabapathy N . The role of dendritic cells in cancer and anti-tumor immunity. Semin Immunol. (2021) 52:101481. doi: 10.1016/j.smim.2021.101481, PMID: 34023170 PMC8545750

[79] Zhou J Nie RC Yin YX Wang Y Yuan SQ Zhao ZH . Genomic analysis uncovers the prognostic and immunogenetic feature of pyroptosis in gastric carcinoma: indication for immunotherapy. Front Cell Dev Biol. (2022) 10:906759. doi: 10.3389/fcell.2022.906759, PMID: 35912105 PMC9328384

[80] Kim MJ Kim K Park HJ Kim GR Hong KH Oh JH . Deletion of PD-1 destabilizes the lineage identity and metabolic fitness of tumor-infiltrating regulatory T cells. Nat Immunol. (2023) 24:148–61. doi: 10.1038/s41590-022-01373-1, PMID: 36577929

[81] Kumar A Das JK Peng HY Wang L Ballard DJ Ren Y . Metabolic fitness of NAC1-deficient Tregs in the tumor microenvironment fuels tumor growth. JCI Insight. (2025) 10. doi: 10.1172/jci.insight.186000, PMID: 39773913 PMC11949012

[82] Kobayashi S Wannakul T Sekino K Takahashi Y Kagawa Y Miyazaki H . Fatty acid-binding protein 5 limits the generation of Foxp3(+) regulatory T cells through regulating plasmacytoid dendritic cell function in the tumor microenvironment. Int J cancer. (2022) 150:152–63. doi: 10.1002/ijc.33777, PMID: 34449874

[83] Zi R Zhao X Liu L Wang Y Zhang R Bian Z . Metabolic-immune suppression mediated by the SIRT1-CX3CL1 axis induces functional enhancement of regulatory T cells in colorectal carcinoma. Advanced Sci (Weinheim Baden-Wurttemberg Germany). (2025) 12:e2404734. doi: 10.1002/advs.202404734, PMID: 39783838 PMC12061293

[84] van Hooren L Handgraaf SM Kloosterman DJ Karimi E van Mil L Gassama AA . CD103(+) regulatory T cells underlie resistance to radio-immunotherapy and impair CD8(+) T cell activation in glioblastoma. Nat cancer. (2023) 4:665–81. doi: 10.1038/s43018-023-00547-6, PMID: 37081259 PMC10212765

[85] Yang L Wang X Wang S Shen J Li Y Wan S . Targeting lipid metabolism in regulatory T cells for enhancing cancer immunotherapy. Biochim Biophys Acta Rev cancer. (2025) 1880:189259. doi: 10.1016/j.bbcan.2025.189259, PMID: 39798823

[86] Song C Ji Y Wang W Tao N . Ginger polysaccharide promotes myeloid-derived suppressor cell apoptosis by regulating lipid metabolism. Phytother research: PTR. (2023) 37:2894–901. doi: 10.1002/ptr.7784, PMID: 36806265

[87] Zhu GQ Tang Z Huang R Qu WF Fang Y Yang R . CD36(+) cancer-associated fibroblasts provide immunosuppressive microenvironment for hepatocellular carcinoma via secretion of macrophage migration inhibitory factor. Cell discovery. (2023) 9:25. doi: 10.1038/s41421-023-00529-z, PMID: 36878933 PMC9988869

[88] Yan Y Huang L Liu Y Yi M Chu Q Jiao D . Metabolic profiles of regulatory T cells and their adaptations to the tumor microenvironment: implications for antitumor immunity. J Hematol Oncol. (2022) 15:104. doi: 10.1186/s13045-022-01322-3, PMID: 35948909 PMC9364625

[89] Wang R Liu Z Fan Z Zhan H . Lipid metabolism reprogramming of CD8(+) T cell and therapeutic implications in cancer. Cancer letters. (2023) 567:216267. doi: 10.1016/j.canlet.2023.216267, PMID: 37315709

[90] Wu D Chen Y . Lipids for CD8(+) TILs: beneficial or harmful? Front Immunol. (2022) 13:1020422. doi: 10.3389/fimmu.2022.1020422, PMID: 36275711 PMC9585227

[91] Chen W Teo JMN Yau SW Wong MY Lok CN Che CM . Chronic type I interferon signaling promotes lipid-peroxidation-driven terminal CD8(+) T cell exhaustion and curtails anti-PD-1 efficacy. Cell Rep. (2022) 41:111647. doi: 10.1016/j.celrep.2022.111647, PMID: 36384131

[92] Zheng M Zhang W Chen X Guo H Wu H Xu Y . The impact of lipids on the cancer-immunity cycle and strategies for modulating lipid metabolism to improve cancer immunotherapy. Acta Pharm Sin B. (2023) 13:1488–97. doi: 10.1016/j.apsb.2022.10.027, PMID: 37139414 PMC10149904

[93] Cui MY Yi X Zhu DX Wu J . The role of lipid metabolism in gastric cancer. Front Oncol. (2022) 12:916661. doi: 10.3389/fonc.2022.916661, PMID: 35785165 PMC9240397

[94] Yang S Sun B Li W Yang H Li N Zhang X . Fatty acid metabolism is related to the immune microenvironment changes of gastric cancer and RGS2 is a new tumor biomarker. Front Immunol. (2022) 13:1065927. doi: 10.3389/fimmu.2022.1065927, PMID: 36591293 PMC9797045

[95] Li Y Zeng Z . Investigating the dysregulation of genes associated with glucose and lipid metabolism in gastric cancer and their influence on immunity and prognosis. BioFactors (Oxford England). (2025) 51:e2138. doi: 10.1002/biof.2138, PMID: 39508713 PMC11681298

[96] Wang Z Chen H Sun L Wang X Xu Y Tian S . Uncovering the potential of APOD as a biomarker in gastric cancer: A retrospective and multi-center study. Comput Struct Biotechnol J. (2024) 23:1051–64. doi: 10.1016/j.csbj.2024.02.015, PMID: 38455068 PMC10918487

[97] Li Y Zheng Y Huang J Nie RC Wu QN Zuo Z . CAF-macrophage crosstalk in tumour microenvironments governs the response to immune checkpoint blockade in gastric cancer peritoneal metastases. Gut. (2025) 74:350–63. doi: 10.1136/gutjnl-2024-333617, PMID: 39537239 PMC11874311

[98] Wang W Gao Y Liu Y Xia S Xu J Qin L . Pan-cancer analysis reveals MTTP as a prognostic and immunotherapeutic biomarker in human tumors. Front Immunol. (2025) 16:1549965. doi: 10.3389/fimmu.2025.1549965, PMID: 40213543 PMC11983653

[99] Hou W Zhao Y Zhu H . Predictive biomarkers for immunotherapy in gastric cancer: current status and emerging prospects. Int J Mol Sci. (2023) 24:15321. doi: 10.3390/ijms242015321, PMID: 37895000 PMC10607383

[100] Kang YK Morita S Satoh T Ryu MH Chao Y Kato K . Exploration of predictors of benefit from nivolumab monotherapy for patients with pretreated advanced gastric and gastroesophageal junction cancer: *post hoc* subanalysis from the ATTRACTION-2 study. Gastric Cancer. (2022) 25:207–17. doi: 10.1007/s10120-021-01230-4, PMID: 34480657 PMC8732926

[101] Kang YK Chen LT Ryu MH Oh DY Oh SC Chung HC . Nivolumab plus chemotherapy versus placebo plus chemotherapy in patients with HER2-negative, untreated, unresectable advanced or recurrent gastric or gastro-oesophageal junction cancer (ATTRACTION-4): a randomised, multicentre, double-blind, placebo-controlled, phase 3 trial. Lancet Oncol. (2022) 23:234–47. doi: 10.1016/S1470-2045(21)00692-6, PMID: 35030335

[102] Rha SY Oh DY Yañez P Bai Y Ryu MH Lee J . Pembrolizumab plus chemotherapy versus placebo plus chemotherapy for HER2-negative advanced gastric cancer (KEYNOTE-859): a multicentre, randomised, double-blind, phase 3 trial. Lancet Oncol. (2023) 24:1181–95. doi: 10.1016/S1470-2045(23)00515-6, PMID: 37875143

[103] Gou M Zhang Y Wang Z Qian N Dai G . PD-1 inhibitor combined with albumin paclitaxel and apatinib as second-line treatment for patients with metastatic gastric cancer: a single-center, single-arm, phase II study. Investigational New Drugs. (2024) 42:171–8. doi: 10.1007/s10637-024-01425-3, PMID: 38347177 PMC10944415

[104] Janjigian YY Shitara K Moehler M Garrido M Salman P Shen L . First-line nivolumab plus chemotherapy versus chemotherapy alone for advanced gastric, gastro-oesophageal junction, and oesophageal adenocarcinoma (CheckMate 649): a randomised, open-label, phase 3 trial. Lancet (London England). (2021) 398:27–40. doi: 10.1016/S0140-6736(21)00797-2, PMID: 34102137 PMC8436782

[105] Butterfield LH Najjar YG . Immunotherapy combination approaches: mechanisms, biomarkers and clinical observations. Nat Rev Immunol. (2024) 24:399–416. doi: 10.1038/s41577-023-00973-8, PMID: 38057451 PMC11460566

[106] Catenacci DV Rosales M Chung HC HY H Shen L Moehler M . MAHOGANY: margetuximab combination in HER2+ unresectable/metastatic gastric/gastroesophageal junction adenocarcinoma. Future Oncol (London England). (2021) 17:1155–64. doi: 10.2217/fon-2020-1007, PMID: 33263418

[107] Luke JJ Bever K Hodi FS Taube J Massey A Yao D . Rationale and feasibility of a rapid integral biomarker program that informs immune-oncology clinical trials: the ADVISE trial. J immunother Cancer. (2025) 13. doi: 10.1136/jitc-2024-011170, PMID: 40389374 PMC12090868

[108] Hegewisch-Becker S Mendez G Chao J Nemecek R Feeney K Van Cutsem E . First-line nivolumab and relatlimab plus chemotherapy for gastric or gastroesophageal junction adenocarcinoma: the phase II RELATIVITY-060 study. J Clin Oncol. (2024) 42:2080–93. doi: 10.1200/JCO.23.01636, PMID: 38723227 PMC11191068

[109] Ji Z Wang X Xin J Ma L Zuo D Li H . Multiomics reveals tumor microenvironment remodeling in locally advanced gastric and gastroesophageal junction cancer following neoadjuvant immunotherapy and chemotherapy. J immunother Cancer. (2024) 12. doi: 10.1136/jitc-2024-010041, PMID: 39653554 PMC11629098

[110] Yang W Zhou M Li G Zhou C Wang L Xia F . Adjuvant chemoradiotherapy plus PD-1 inhibitor for pN3 gastric cancer: a randomized, multicenter, Phase III trial. Future Oncol (London England). (2024) 20:3389–96. doi: 10.1080/14796694.2024.2421156, PMID: 39545610 PMC11776863

[111] Janjigian YY Kawazoe A Bai Y Xu J Lonardi S Metges JP . Pembrolizumab plus trastuzumab and chemotherapy for HER2-positive gastric or gastro-oesophageal junction adenocarcinoma: interim analyses from the phase 3 KEYNOTE-811 randomised placebo-controlled trial. Lancet (London England). (2023) 402:2197–208. doi: 10.1016/S0140-6736(23)02033-0, PMID: 37871604

[112] Bagaev A Kotlov N Nomie K Svekolkin V Gafurov A Isaeva O . Conserved pan-cancer microenvironment subtypes predict response to immunotherapy. Cancer Cell. (2021) 39:845–65.e7. doi: 10.1016/j.ccell.2021.04.014, PMID: 34019806

[113] Zeng D Wu J Luo H Li Y Xiao J Peng J . Tumor microenvironment evaluation promotes precise checkpoint immunotherapy of advanced gastric cancer. J immunother Cancer. (2021) 9. doi: 10.1136/jitc-2021-002467, PMID: 34376552 PMC8356190

[114] Li S Yu W Xie F Luo H Liu Z Lv W . Neoadjuvant therapy with immune checkpoint blockade, antiangiogenesis, and chemotherapy for locally advanced gastric cancer. Nat Commun. (2023) 14:8. doi: 10.1038/s41467-022-35431-x, PMID: 36596787 PMC9810618

[115] Shitara K Ajani JA Moehler M Garrido M Gallardo C Shen L . Nivolumab plus chemotherapy or ipilimumab in gastro-oesophageal cancer. Nature. (2022) 603:942–8. doi: 10.1038/s41586-022-04508-4, PMID: 35322232 PMC8967713

[116] Shen L Zhang Y Li Z Zhang X Gao X Liu B . First-line cadonilimab plus chemotherapy in HER2-negative advanced gastric or gastroesophageal junction adenocarcinoma: a randomized, double-blind, phase 3 trial. Nat Med. (2025) 31:1163–70. doi: 10.1038/s41591-024-03450-4, PMID: 39843940

[117] Chen L Deng J . Role of non-coding RNA in immune microenvironment and anticancer therapy of gastric cancer. J Mol Med (Berlin Germany). (2022) 100:1703–19. doi: 10.1007/s00109-022-02264-6, PMID: 36329206

[118] Jing Q Yao H Li H Yuan C Hu J Zhang P . A novel RNA modification prognostic signature for predicting the characteristics of the tumor microenvironment in gastric cancer. Front Oncol. (2023) 13:905139. doi: 10.3389/fonc.2023.905139, PMID: 36874129 PMC9978099

[119] Yang P Yang W Wei Z Li Y Yang Y Wang J . Novel targets for gastric cancer: The tumor microenvironment (TME), N6-methyladenosine (m6A), pyroptosis, autophagy, ferroptosis and cuproptosis. Biomed pharmacother = Biomed pharmacotherapie. (2023) 163:114883. doi: 10.1016/j.biopha.2023.114883, PMID: 37196545

[120] He X Guan XY Li Y . Clinical significance of the tumor microenvironment on immune tolerance in gastric cancer. Front Immunol. (2025) 16:1532605. doi: 10.3389/fimmu.2025.1532605, PMID: 40028336 PMC11868122

[121] Chang J Wu H Wu J Liu M Zhang W Hu Y . Constructing a novel mitochondrial-related gene signature for evaluating the tumor immune microenvironment and predicting survival in stomach adenocarcinoma. J Trans Med. (2023) 21:191. doi: 10.1186/s12967-023-04033-6, PMID: 36915111 PMC10012538

[122] Su C Yu R Hong X Zhang P Guo Y Cai JC . CXCR4 expressed by tumor-infiltrating B cells in gastric cancer related to survival in the tumor microenvironment: an analysis combining single-cell RNA sequencing with bulk RNA sequencing. Int J Mol Sci. (2023) 24:12890. doi: 10.3390/ijms241612890, PMID: 37629071 PMC10454711

[123] Pang MJ Burclaff JR Jin R Adkins-Threats M Osaki LH Han Y . Gastric organoids: progress and remaining challenges. Cell Mol Gastroenterol hepatol. (2022) 13:19–33. doi: 10.1016/j.jcmgh.2021.09.005, PMID: 34547535 PMC8600088

[124] Bin YL Hu HS Tian F Wen ZH Yang MF Wu BH . Metabolic reprogramming in gastric cancer: trojan horse effect. Front Oncol. (2021) 11:745209. doi: 10.3389/fonc.2021.745209, PMID: 35096565 PMC8790521

[125] Chen X Zhou B Wang S Jiang X Ping Y Xia J . Intestinal metaplasia key molecules and UPP1 activation via Helicobacter pylori/NF-kB: drivers of Malignant progression in gastric cancer. Cancer Cell Int. (2024) 24:399. doi: 10.1186/s12935-024-03598-6, PMID: 39695769 PMC11657005

[126] Shang Z Ma Z Wu E Chen X Tuo B Li T . Effect of metabolic reprogramming on the immune microenvironment in gastric cancer. Biomed pharmacother = Biomed pharmacotherapie. (2024) 170:116030. doi: 10.1016/j.biopha.2023.116030, PMID: 38128177

[127] Jiang T Zhang J Zhao S Zhang M Wei Y Liu X . MCT4: a key player influencing gastric cancer metastasis and participating in the regulation of the metastatic immune microenvironment. J Trans Med. (2025) 23:276. doi: 10.1186/s12967-025-06279-8, PMID: 40045374 PMC11884109

[128] Liu K Yuan S Wang C Zhu H . Resistance to immune checkpoint inhibitors in gastric cancer. Front Pharmacol. (2023) 14:1285343. doi: 10.3389/fphar.2023.1285343, PMID: 38026944 PMC10679741

[129] Liu J Yuan Q Guo H Guan H Hong Z Shang D . Deciphering drug resistance in gastric cancer: Potential mechanisms and future perspectives. Biomed pharmacother = Biomed pharmacotherapie. (2024) 173:116310. doi: 10.1016/j.biopha.2024.116310, PMID: 38394851

[130] Zhang Y Yang Y Chen Y Lin W Chen X Liu J . PD-L1: Biological mechanism, function, and immunotherapy in gastric cancer. Front Immunol. (2022) 13:1060497. doi: 10.3389/fimmu.2022.1060497, PMID: 36505487 PMC9729722

[131] Yang T Guo L . Advancing gastric cancer treatment: nanotechnology innovations and future prospects. Cell Biol toxicol. (2024) 40:101. doi: 10.1007/s10565-024-09943-9, PMID: 39565472 PMC11579161

[132] Keshavjee SH Moy RH Reiner SL Ryeom SW Yoon SS . Gastric cancer and the immune system: the key to improving outcomes? Cancers. (2022) 14:5940. doi: 10.3390/cancers14235940, PMID: 36497422 PMC9739366

