# Correction: The impact of aberrant lipid metabolism on the immune microenvironment of gastric cancer: a mini review

**DOI:** 10.3389/fimmu.2025.1761409

**Published:** 2025-12-12

**Authors:** Shuangyu Chen, Wenqian Chen, Tinghui Xu, Jiayang Li, Jianghao Yu, Yibo He, Shengliang Qiu

**Affiliations:** 1 The First Affiliated Hospital of Zhejiang Chinese Medical University (Zhejiang Provincial Hospital of Chinese Medicine), Hangzhou, Zhejiang, China; 2 School of Medical Technology and Information Engineering, Zhejiang Chinese Medical University, Hangzhou, Zhejiang, China

**Keywords:** gastric cancer, lipid metabolism, tumor immune microenvironment, CD8+ T cells, tumor-associated macrophages, immunotherapy resistance, fatty acid oxidation, immune checkpoint blockade

Author “Shengliang Qiu” was erroneously missed as corresponding author. The correct corresponding authors are: “Shengliang Qiu and Yibo He”.

The original version of this article has been updated.

